# Mapping geochemical distribution, toxicity and ecological risk indices of potentially toxic elements in cultured fish and surface water (Blacksea catchment/Türkiye)

**DOI:** 10.1007/s10534-025-00785-4

**Published:** 2026-01-20

**Authors:** Mustafa Türkmen, Erkan Kalıpcı, Mehmet Ali Dereli, Hüseyin Cüce, Aysun Türkmen

**Affiliations:** 1 Department of Biology, Giresun University, Giresun City, Turkey; 2 Deparment of Geomatics Engineering, Giresun University, Giresun City, Turkey; 3 Deparment of Environmental Engineering, Giresun University, Giresun City, Turkey; 4 Deparment of Chemistry, Giresun University, Giresun City, Turkey

**Keywords:** Multi-source data, PTEs bioaccumulation, Geochemical distribution map, Public health risk, *Rainbow trout*

## Abstract

In this study, the bioaccumulation levels, the geochemical distributions and the ecotoxicological risk levels of potential toxic elements (PTEs: Al, Cr, Mn, Fe, Co, Ni, Cu, Zn, As, Se, Cd, Hg and Pb) were determined in water and fish (*Rainbow trout)* collected from 15 different ponds in the Black Sea coastal basin. Among the PTEs in muscle tissue, Fe was determined to be at the highest level, while Cd and Co were at the lowest level. It was observed that As and Mn were above the maximum permissible levels. Water Quality Index (WQI) values were excellent at all stations, and no pollution levels were detected that would threaten human health according to the Heavy Metal Pollution Index (HPI) and the Heavy Metal Evaluation Index. The metal pollution index level in fish produced in inland waters in the study area was less than 2 (0.78), indicating that there may not be a potential health risk due to the bioaccumulation pattern. However, the target hazard coefficient (THQ) for As was > 1 at all stations except S1 and S15, and the hazard index was > 1 at all stations except S1, suggesting the possibility of non-carcinogenic adverse health effects. When evaluated in terms of total cancer risk level, it suggests that there may be a cancer risk due to metal accumulation in all stations except S1.

**Supplementary Information:**

The online version contains supplementary material available at 10.1007/s10534-025-00785-4.

## Introduction

Because of their widespread presence, non-degradability, availability, bioaccumulation, and high toxicity, potential trace/toxic elements (PTEs) have emerged as one of the biggest global environmental concerns in recent decades (Ouro-Sama et al. 2025). The health of humans and other living organisms, including those living both in water and on land, is affected by the contamination of PTEs in water and soil. Toxic metal poisoning is associated with various health problems, including various types of cancer, kidney and liver failure, dermatological disorders, and cognitive impairments (Manna and Firdous 2025). The production of solid and liquid sludges, which are characterised by high concentrations of toxic heavy metals, such as Zinc (Zn), Lead (Pb), Aluminum (Al), Cadmium (Cd), Nickel (Ni), Iron (Fe), Manganese (Mn), and Arsenic (As), poses a significant threat to coastal waters, soil, plants and food sources (Yüksel et al. 2025; Sama et al. 2024; Muhammad et al. 2024; Ustaoğlu et al. 2024; Muhammad 2023; Okereafor et al. 2020; Temizer et al; 2018; Skoczko and Szatyłowicz 2018). Arsenic, cadmium, lead, and mercury are among the other elements that are non-essential and have no beneficial roles in humans, animals, or plants (Antoine et al. 2012; Khan et al. 2015; Önel et al. 2025a; Cüce et al. 2025, 2022a, 2022b; Kalipci et al. 2023; Aydın and Güner 2020). Human exposure to heavy metals can often come from dietary sources, medications, environmental factors, or occupational and recreational activities. Edible vegetables, fruits, and seafood account for approximately 90% of total exposure, while the remaining 10% occurs thru skin contact and inhalation of contaminated dust. Toxic metal poisoning in aquatic organisms or vegetation has the potential to bioaccumulate and persist in the food chain, eventually spreading to humans (Xu et al. 2024; Luo et al. 2014). PTEs enter the food chain of fish through respiration, adsorption, and ingestion, accumulating in soft tissues. Due to their high toxicity, they lead to serious health problems and threaten ecological life (Ustaoğlu et al. 2024; Ustaoğlu and Yüksel 2024; Varol and Kaçar 2023; Kalipci et al. 2023; Kalipci 2019). Despite all these adverse ecotoxicological effects, fish has become a pervasive global phenomenon, with this seafood product being one of the most widely consumed worldwide due to their content of unsaturated omega fatty acids, microelements, vitamins, and high protein levels (Kaçar 2024; Varol 2022; Varol and Sünbül, 2020). Among the species cultivated worldwide, *Rainbow trout* (Oncorhynchus mykiss) ranks 15th and is among the leading species of farmed trout. In global *Rainbow trout* aquaculture, Chile ranks first with 326,054 tons, followed by Iran with 193,852 tons, and Türkiye with 165,683 tons. Türkiye’s trout production has grown 3.75 times over the past 20 years, reaching 167,286 tons/year, becoming a significant food production industry (FAO 2023; Çakmak et al. 2024). The *Rainbow trout* produced is shipped through international trade channels as a widely consumed food product to many countries, especially in Europe. In recent years, in order to counter the decline in wild fish stocks and to meet the increasing demand for fish-based protein due to population growth, *Rainbow trout* production has taken the lead in aquaculture development in Türkiye’s Black Sea Region. With freshwater resources, dam lakes, and marine environments offering highly suitable ecological conditions for trout production, the region has become an attractive production hub. The fact that The Black Sea is responsible for the supply of 76% of Türkiye's fish production (Sirkecioğlu 2002), that the Black Sea Region is the richest region in terms of the number of pond farm enterprises (266 units), and that there is no comprehensive research conducted in the region on this subject were the main reasons for selecting pond farms in the Black Sea Region as the study area (Tüik 2022; Çakmak et al. 2024). On the other hand, chemical pollution in the Black Sea such as petroleum, untreated wastewater discharges, PTEs, and pesticides are harming biological life, causing structural damage in fish at the cellular and molecular level, and leading to PTEs toxicity in humans through the consumption of fish, which constitute a critical link in the food chain (Önel et al. 2025b; Kalipci and Namal 2018; Verep and Mutlu 2022; Turkmen and Budur 2018; Fulladosa et al. 2006; Türkmen et al. 2018; Özdemir et al. 2012; Kalipci et al. 2011; Kalay et al. 2004; Kalipci et al. 2010; Önel et al. 2025c). Prolonged exposure of fish to PTEs causes toxic effects in fish, and the consumption of these fish by humans may lead to cardiovascular diseases, nervous system disorders, reproductive and hematological effects, developmental anomalies, and kidney and liver damage (Singh and Kumar 2017; Türkmen and Öğütcü 2020; Qu et al. 2018). In recent years, PTEs have been distributed in various ways and bioaccumulate especially in fish and threaten public health. PTEs such as Cd, Pb, Hg, Ni, As and Cr have been reported to cause toxic effects especially in children and pregnant women because they can be partially excreted through urine and feces when daily tolerable intake limits are exceeded. Toxic metals poses a threat to health in several ways, such as disrupting intracellular calcium balance, causing oxidative stress, disrupting cell signaling processes, and leading to epigenetic effects. Fish directly absorb free ions dissolved in water through their skin, gills, and digestive systems (Qu and Zheng 2024; Li et al. 2009). Therefore, it is emphasized that studies aimed at determining the concentration of PTEs in marine organisms are of great global importance and should be conducted periodically to ensure food safety and consumer awareness (Demir et al. 2024, 2021; Töre et al. 2021; Dirican et al. 2013; Kalipci et al. 2023; Abbas et al. 2024). The ecological hazards of *Rainbow trout* and the elevated PTEs levels in the waterways of Türkiye's Eastern Black Sea Region were to be examined for this study. The following are the study's objectives:

I. This study's first goal is to determine the levels of PTEs bioaccumulation in rainbow trout and water. II. To assess the health hazards of metal toxicity, a number of impact assessment tools were used for ecotoxicological analysis, including metal pollution index (MPI), bioconcentration factor (BCF), target hazard coefficient (THQ), hazard index (HI), carcinogenic risk (CR), total cancer risk (TCR), estimated daily intake rate (EDI), and various ecotoxicological indices such as water quality indices (WQI), heavy metal pollution index (HPI), and heavy metal assessment index (HEI). III. Using some important statistical indicators such as Pearson correlation index (PCI), Principal component analysis (PCA) and cluster analysis (CA); to measure the linear relationship between potentially toxic elements, to reveal their principal components using linear combinations of variables in the PTEs data set, and to perform detailed statistical analysis by clustering the elements according to their similarity to others, IV. To map the accumulation of PTEs in water and *Rainbow trout* in fish farms in the Eastern Black Sea region using Geographic Information Systems (GIS) and to show their geochemical distribution. As far as we know from the literature review, this is the first large-scale study on the contamination of *Rainbow Trout* and water produced in 15 different fish farms in the Eastern Black Sea region of Türkiye with PTEs and its ecological and public health risks.

## Material and methods

### Study zone and collection of fish and surface water samples

**Fig. 1:**
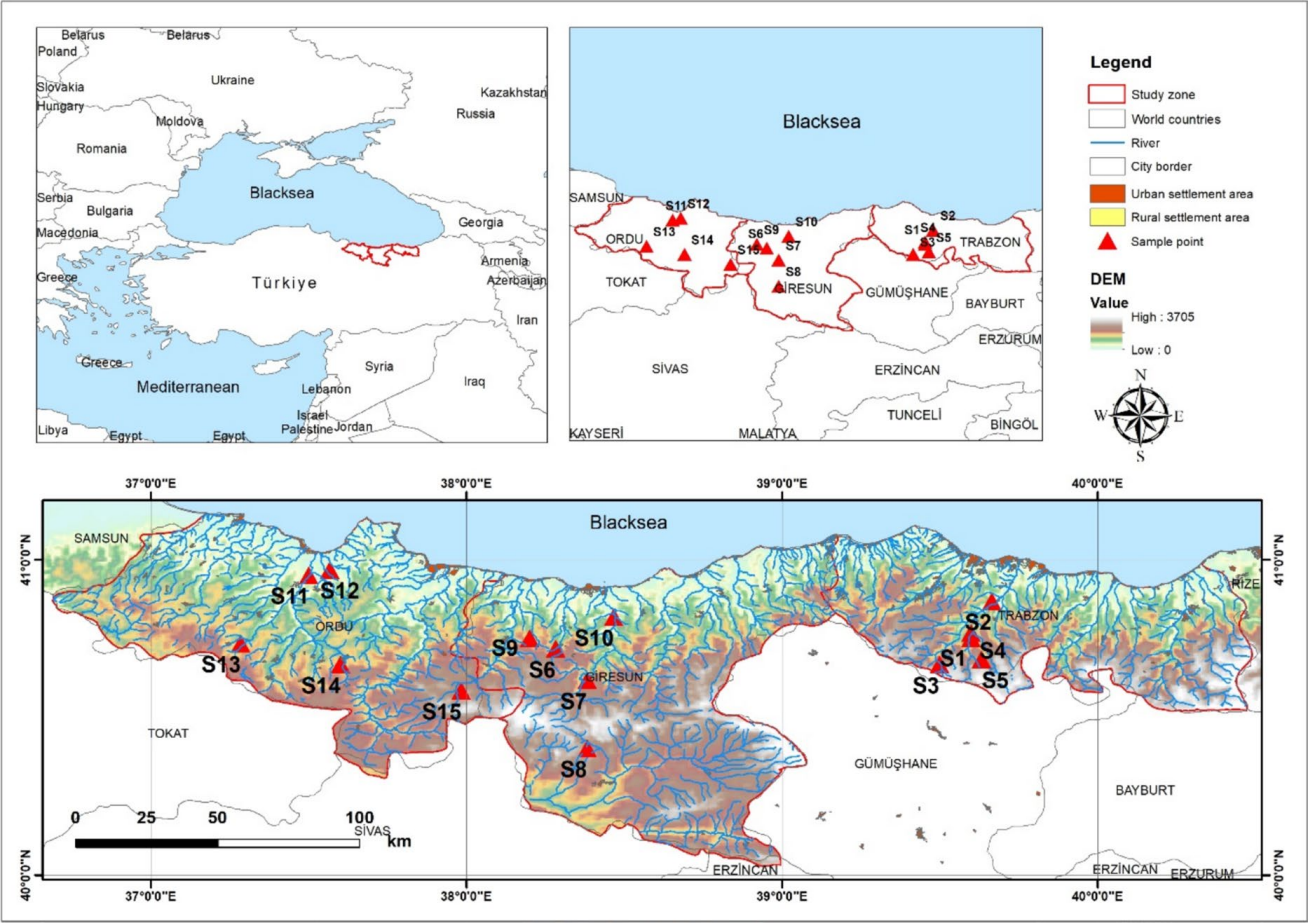
Fish and surface water sampling stations and study zone

The Black Sea is a semi-enclosed sea, with the world's oceans accessible only via the narrow Bosphorus. The region's distinctive environmental characteristics, including the perpetual stratification between oxygenated surface waters and oxygen-free deep layers, contribute to the establishment of a diverse and sensitive marine ecosystem. As illustrated in Fig. 1, It is bordered by six countries—Bulgaria, Georgia, Romania, Russia, Turkey, and Ukraine—and influenced by 17 basin countries, 13 capital cities, and ~ 160 million people. The Turkish Black Sea coast, which is approximately 1,695 km long, extends from the Bulgarian border in the west to the Georgian border in the east (Bisinicu and Harcota 2025; Bosneagu et al. 2018; Manev et al. 2020). A detailed analysis was conducted for a total of 14 PTEs in water and *Rainbow trout* (*Oncorhynchus mykiss*) samples collected from 15 fish farms located in the Eastern Black Sea Region of Türkiye. Since the fish were sampled by farm personnel during harvesting, ethical approval was not required for this study. The stations from which water and *Rainbow trout* samples were collected, along with the study area, are shown in Fig. 1. Given the potential threat of water pollution to the aquaculture industry and the need to ensure the safety of farmed fish, the primary objective was to examine the dependence of bioaccumulation on the food chain and environmental heavy metal concentrations in the water at each station collecting rainbow trout, the primary freshwater fish species farmed in the Black Sea region.

### Experimental design of samples for metal analysis in surface water and fish

Four water samples were taken from each fish farm in 1 L polyethylene bottles and 1% HNO_3_ (Merck) with 65% purity was dropped on it. It was then passed through a 0.45 µ filter, placed in 50 ml glass bottles and kept in the refrigerator until analysis (Cüce et al. 2025, 2022a; Cataldo et al. 2001). Ten fish samples were taken from each fish farm and brought to the laboratory in ice-protected containers. The fish samples weighed between 200 and 300 g and were between 26 and 30 cm in length. Then, muscle samples taken from ten fish from each station were completely homogenized. For each station, four 0.5 g subsamples were taken from homogenized samples and stored at −18 °C for five days until further analysis. Tissue samples were digested in Teflon vessels with a combination of concentrated grade HNO_3_ (%65) and H_2_O_2_ (%30) (7:1) in accordance with (HPR-FO-67) temperature and pressure profile using a microwave oven (Milenstone Start D SK-10) and passed through a 0.45 µm filter. All laboratory equipment were before use. acid washed (2 M HNO_3_ in for 48 h) to prevent any potential contamination. To analyze the metal concentration in fish samples, a standard reagent (Merck, Germany) prepared using the same acid matrix as the samples was used. All equipment was rinsed five times with deionized water and made ready for use. A certified lobster hepatopancreas (National Research Council of Canada) from Tort-2 was used as the calibration verification standard. For water samples, an UME CRM 1201 spring water was used the verification standard. The recovery rates of the certified reference materials used in the study are presented in Table S1 (see the Supplementary Material File). Both fish and water samples were analyzed three times for each metal by ICP-MS (Agilent 7700x) and averages were utilised as a statistical measure (Kalipci et al. 2023; Töre et al. 2021).

### Assessment method

The public health impacts of *Rainbow trout* were investigated using PTEs concentrations determined in *Rainbow trout* and multidimensional health risk indices (MPI, BCF, THQ, HI, CR, TCR and EDI). In water sampling, ecotoxicological indices such as Water quality indices (WQI), Heavy metal pollution index (HPI) and Heavy metal evaluation index (HEI) and health risk assessments based on toxicological parameters were calculated and the results were evaluated. In 15 different fish farms, the average potential toxic elements (PTEs) measurement results of water and muscle of fish (Table S2 and S3), and also the formulas employed in these calculations (Table S4) are provided in the Supplementary Material File.

### Statistical and geospatial analyses

Multivariate statistical methods have been widely used in studies with potentially toxic elements in water and food products in recent years (Islam et al. 2025; Hasan et al. 2020; Dereli et al. 2024; Varol et al. 2025). Therefore, reducing the data set and determining whether there was any possible association between the harmful elements under investigation were the goals of PCA. Kaiser–Meyer–Olkin (KMO) and Bartlett's tests of sphericity were used to assess the data's relevance—that is, to see whether any correlations existed between the variables—before the principal component analysis (PCA) was carried out (Cüce et al. 2022c, 2022d). The results of Bartlett's sphericity test should have a p-value less than 0.001, and it is generally agreed that KMO values more than 0.5 are appropriate for PCA (Liu et al. 2003). Considering the KMO value (0.7) and Bartlett sphericity test value (p = 0) of the current study, the data set appears to be suitable for PCA. Statistical analysis of the data was done with the SPSS 22 (Statistical Package for Social Sciences) package program. The Shapiro–Wilk and Kolmogorov–Smirnov tests were used to determine whether the distribution of the samples was normal. One-way analysis of variance (ANOVA) was used to assess the differences between groups with a normal distribution, and Kruskall-Wallis analysis of variance was used to assess the differences between groups without a normal distribution. Geochemical spatial analyses were performed using ArcGIS 10.8 software. Relationships between PTEs concentrations in fish muscles and water were evaluated with Pearson Correlation index (PCI). In addition, Cluster analysis (CA) and Principal component analysis (PCA) were applied.

## Results and discussion

### PTEs value levels detected in fish samples

The average concentrations of PTEs detected in rainbow trout muscles collected from 15 pond farms in the study region which is widely consumed and economically valuable, are presented in Table S2. The mean PTEs levels in the *Rainbow trout* are listed in the following order: Fe (55.2 mg/kg) > Zn (37.9 mg/kg) > Al (18.2 mg/kg) > Cu (2.32 mg/kg) > Mn (1.83 mg/kg) > As (1.38 mg/kg) > Se (0.76 mg/kg) > Cr (0.26 mg/kg) > Ni (0.25 mg/kg) > Pb (0.14 mg/kg) > Hg (0.08 mg/kg) > Co (0.02 mg/kg) > Cd (0.02 mg/kg) ww. Similar results have been reported for other fish species in studies conducted by many researchers (Peñaloza et al. 2023; Türkmen and Öğütcü 2020; Kalipci et al. 2023; Türkmen et al. 2008). Başkaya et al. (2023) reported that they observed that the PTEs contents of *Rainbow trout* in different growing conditions were Zn > Fe > Se > As in terms of size.

Comparison of the average PTEs levels (mg/kg) detected in the study with MPLs for fish muscle tissues and other study results are given in Table 1. The maximum permissible level of As in *Rainbow trout* muscle was found to exceed the limit values of 0.1 mg/kg and 0.6 mg/kg, respectively, according to the quality standards set by the Chinese Ministry of Health and the National Organisation for Iranian Standards. This excess was particularly evident at stations S3, S4 and S5, which are located in areas with mining contamination, suggesting that this may have led to long-term bioaccumulation. A significant portion of Türkiye’s Cu, Zn and Pb reserves are located in the Eastern Black Sea Region. Long-term exposure to low levels of arsenic leads to the accumulation of arsenic in liver and kidney tissues in freshwater fish. Bioaccumulation of arsenic in fish has been found to have significant effects on various physiological systems, including reproduction, growth, gene expression, ion control, histopathology and immune system function. It has also been reported that rainbow trout exposed to arsenic exhibit increased production of stress response proteins (Singh and Sharma 2024; Garai et al. 2021). Harkabusová et al. (2009) reported that As content in *Rainbow trout (Oncorhynchus mykiss*) varied between 0.72 and 2.23 mg/kg in muscle tissue, Başkaya et al. (2023) reported that As content in trout varied between 0.42 and 0.56 mg/kg. The mean Mn concentrations (1.83 mg/kg) in *Rainbow trout* were found to be above the MPLs (1 mg/kg) set by the World Health Organisation (Mokhtar et al. 2009). Zn (37.9 mg/kg) exceeded FAO limits (30 mg/kg), while Cu (2.32 mg/kg) remained well below WHO/FAO thresholds (30 mg/kg) (FAO 1983; Mokhtar et al. 2009). Freshwater fish exposed to copper in the water may develop an oxidative stress response. Fish with chronic copper toxicity may exhibit poor development, shorter lifespans, reduced immune responses, and reproductive problems (Yacoub and Gad 2012). Cr and Ni concentration values were higher than the legal permissible value determined by Iranian Standard National Organization (ISNO 2012) and lower than the legal permissible value determined by World Health Organisation (Mokhtar et al. 2009). The mean Al concentrations (18.2 mg/kg) in *Rainbow trout* were above the MPLs (0.10 mg/kg) set by the Chinese Ministry of Health (MHPRC 2013). Cd, Cu, Fe and Pb concentrations in *Rainbow trout* were found to be well below the legal permissible values (Table 1). The average Hg level in the fish in this study was found to be 0.08 mg/kg. According to Turkish Food Codex, FAO, and EC, there should be no more than 0.5, 0.5, and 0.5 mg/kg of Hg in fish, in turn. In this study, it was determined that the average Hg levels were within the permitted MPLs.

**Table 1:** Comparison of the mean levels of PTEs (mg/kg) detected in this study with maximum permissible limits (MPLs) for fish muscle tissues and other study results

Maximum permissible limits (MPLs)	As	Cd	Al	Co	Cr	Cu	Fe	Mn	Ni	Pb	Hg	Zn	References
Mean value detected	1.38	0.02	18.2	0.02	0.26	2.32	55.26	1.83	0.25	0.14	0.08	37.9	This study
Turkish Food Codex	–	0.05	–	–	–	–	–	–	–	0.3	0.50	–	Anonymous (2011)
Food Standards AU New Zealand	2	–	–	–	–	–	–	–	–	0.5	–	–	FSANZ (2013)
European Commission	–	0.05	–	–	–	–	–	–	–	0.3	0.50	–	EC (2006)
World Health Organisation	–	1.00	–	–	50	30	100	1	0.5–1	2.0	–	100	Mokhtar et al. (2009)
Codex Alimentarius Commission	–	–	–	–	–	–	–	–	–	0.3	–	–	WHO/FAO (2015)
Chinese Health Ministry	0.1	0.1	0.10	–	2.0	–	–	–	–	0.5	–	–	MHPRC (2013)
Food and Agriculture Organization	–	0.5	–	–	–	30	–	–	–	–	0.50	30	FAO (1983)
Iranian Standard National Organization	0.6	–	–	–	0.03	–	–	–	0.05	0.3	–	–	ISNO (2012)
*Study zone*													
Blacksea-Türkiye	–	0.0060	–	–	–	0.16	–	–	–	0.028	0.0069	2.93	Bat et al. (2024)
Fish farms Tishgo River-Peru	0.11	–	–	–	–	0.21	–	–	–	0.24	–	2.51	Peñaloza et al. (2023)
Adana-Kozan-Türkiye	0.56	–	–	–	–	–	3.69	–	–	–	–	4.69	Başkaya et al. (2023)
Konya-Türkiye	0.42	0.011	1.25	–	0.30	–	1.97	0.46	0.42	–	–	2.46	Almafrachi et al. (2025)
Kayseri-Türkiye	0.055	–	8.05	0.01	0.14	-	3.29	–	0.41	0.195	0.183	0.39	Tokalioglu et al. (2023)
Fish farms-Polish	0.36	–	0.06	–	–	–	–	–	–	1.19	–	–	Jarosz‐Krzemińska et al. (2021)
Fish farms-Türkiye	–	–	–	0.75	0.56	0.40	8.300	0.78	1.05	–	–	3.51	Varol et al. (2017)
Fish farms-Bangladesh	0.033	0.049	–	–	0.65	–	–	–	0.08	0.53	–	13.31	Alam et al. (2023)
Fish farms-Pakistan	1.28	2.2	–	–	2.37	1.39	–	4.79	0.46	4.46	–	94.05	Habib et al. (2024)
Fish farms-China	0.039	0.003	–	–	–	0.25	–	–	–	0.017	–	5.88	Yu et al. (2013)

### PTEs value levels detected in surface water

In the water samples collected from 15 pond farms, the mean PTEs levels in the water are listed in the following order: Zn > Al > Fe > Hg > Cu > Pb > Mn > Co > As > Ni > Se > Cr > Cd with the levels of 113.7 > 29.9 > 7.01 > 1.79 > 0.70 > 0.30 > 0.21 > 0.21 > 0.20 > 0.19 > 0.16 > 0.10 > 0.05 µg/l, respectively (Table S3). Peñaloza et al. (2023) reported that PTEs concentrations in water were measured in decreasing order as Zn > Pb > As > Cu. Although As, Fe and Mn concentrations appear low in surface water, their accumulation in fish muscle tissue is probably related to bioaccumulation or trophic transfer (Xia et al. 2019; Jiang et al. 2018). Al, Fe and Mn are the three elements with the highest levels due to the geogenic structure of the region. Therefore, these elements were naturally detected in the water and *Rainbow trout*. The presence of arsenic is thought to be of lithogenic and anthropogenic origin.

**Fig. 2:**
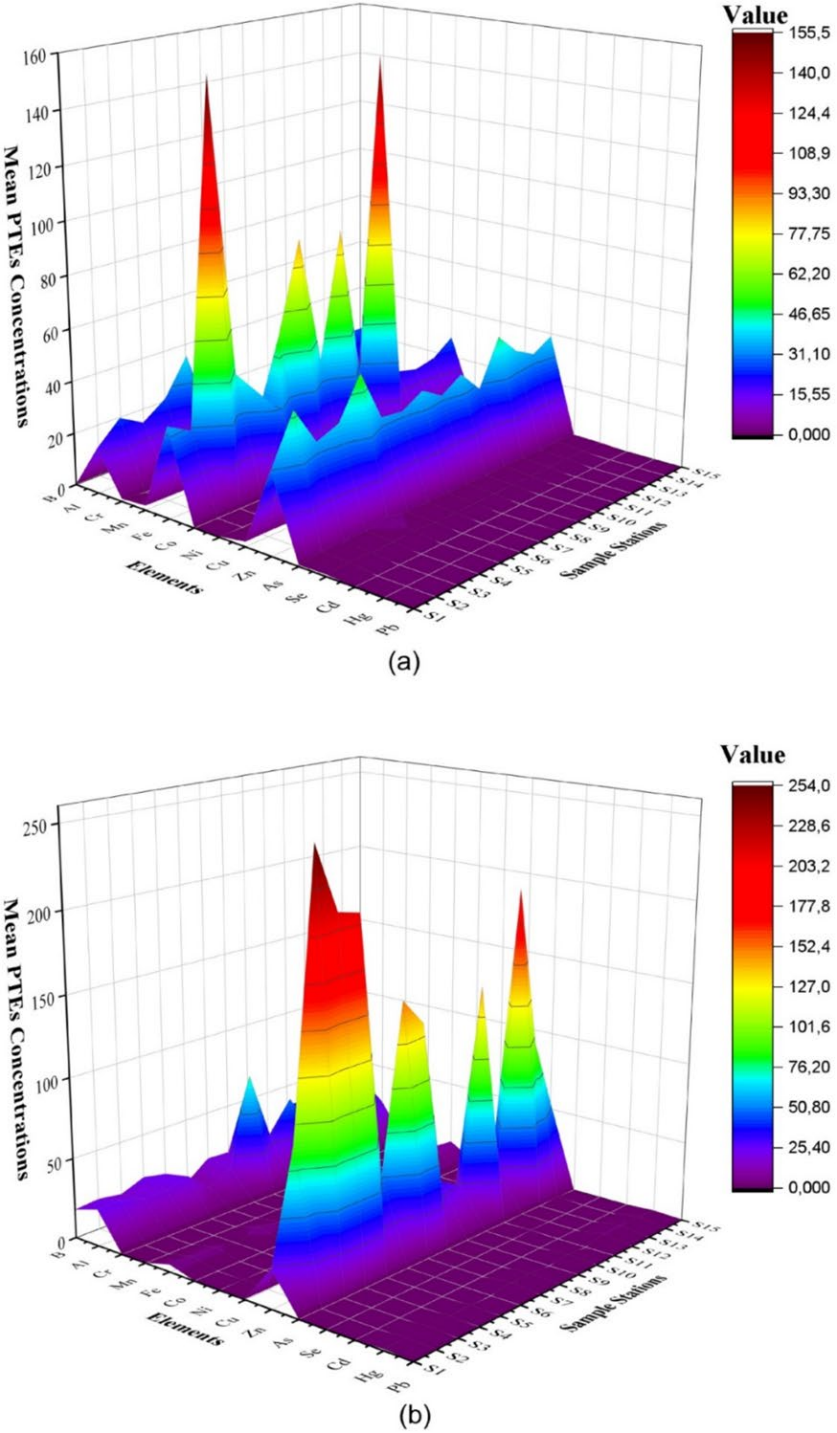
Distribution of PTEs in *Rainbow trout* **a** (mg/kg wet weight) and water **b** (µg/l) by sampling stations

Figure 2 shows the combined average distribution of PTEs in fish and water based on the sampling stations. *Rainbow trout* samples often had the highest average levels of PTEs for Fe, Al, and Zn (Fig. 2). Similar to the levels found in the Rainbow trout samples, the greatest average PTEs levels were found in water samples for Al, Zn, and Fe (Tables S1 and S2).

### Evaluation of PTEs accumulation geochemical map

**Fig. 3:**
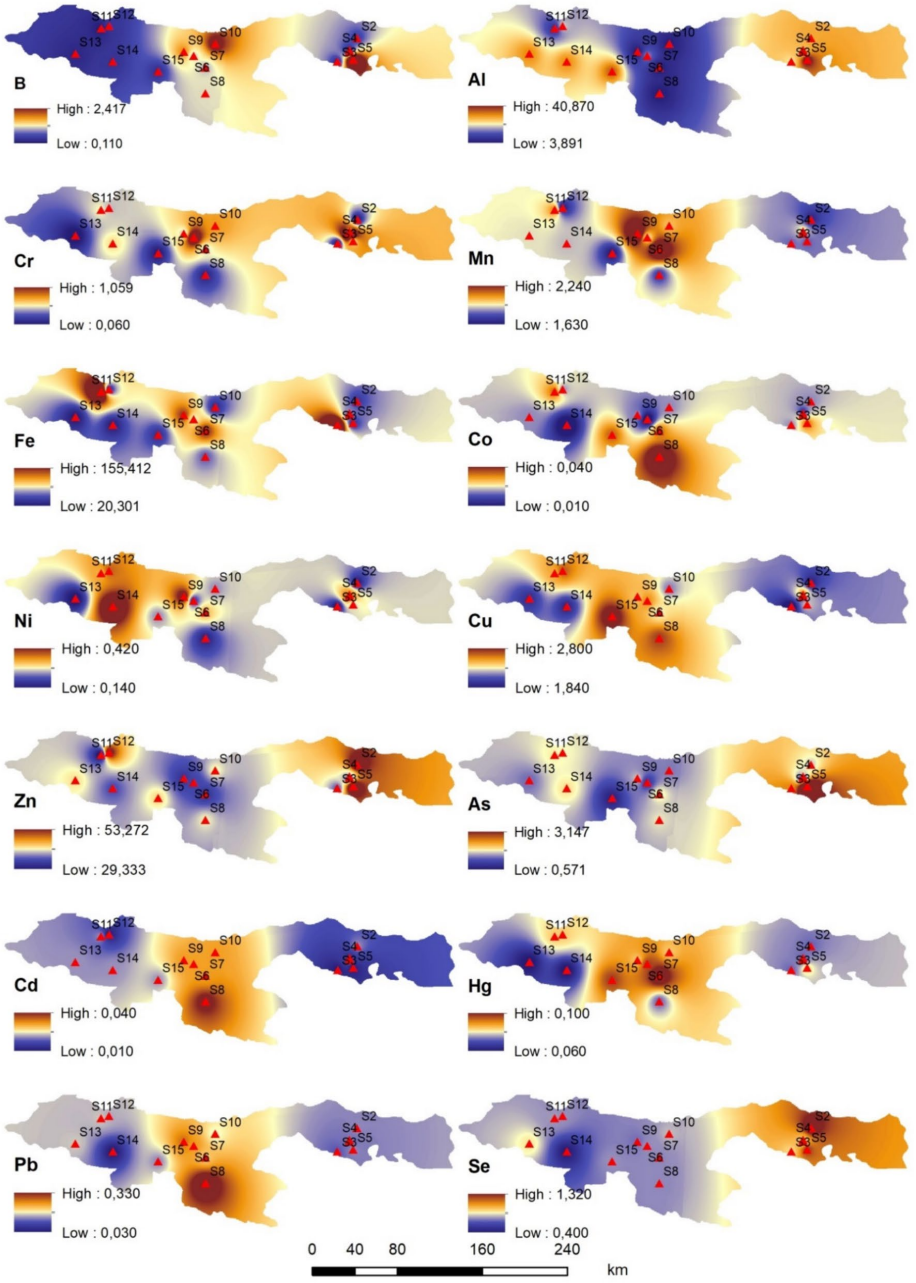
Geochemical distribution analysis map of PTEs in *Rainbow trout* (mg/kg)

**Fig. 4:**
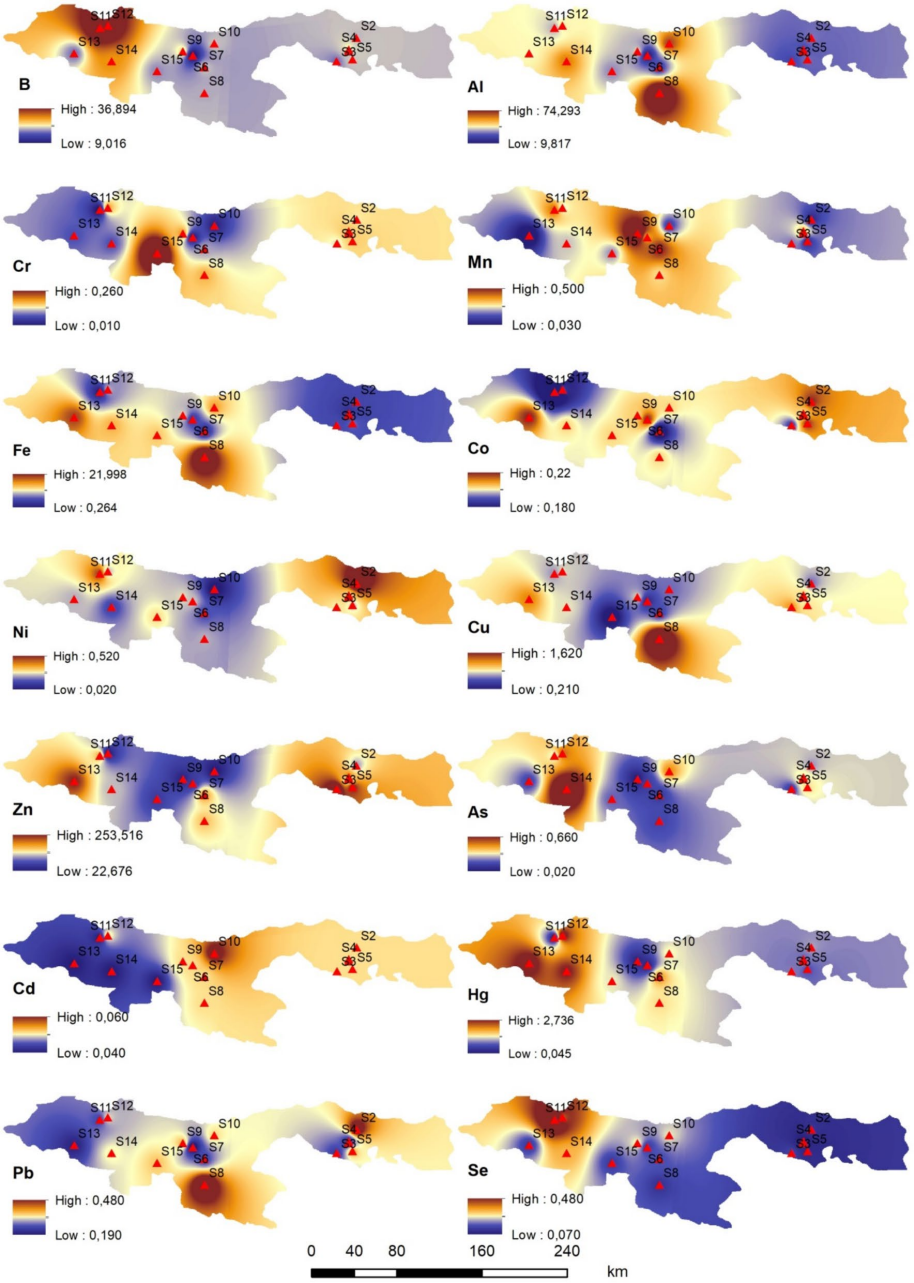
Geochemical distribution analysis map of PTEs in water (µg/l)

The geochemical distribution analysis maps showing the accumulation of PTEs (Al, Cr, Mn, Fe, Co, Ni, Cu, Zn, As, Se, Cd, Hg, and Pb) in *Rainbow trout* and water from 15 pond farms located in the Black Sea Region are presented in Fig. 3 and 4. In this study, in order to perform the geochemical distribution analysis, ArcGIS 10.8 software was used, and mapping process was conducted through the establishment of a continuous surface for the designated study area, employing the inverse distance weighted (IDW) approach, which is one of the interpolation techniques. This method, which has been increasingly applied in many recent studies (Kalipci et al. 2024a, 2024b; Dereli et al. 2024; Cüce et al. 2025).

It is predicated on the idea that there exist connections between the geographically dispersed geochemical sampling locations in the research area (Tercan and Dereli 2020; Shukla et al. 2020). As shown in Fig. 3, the highest metal levels in *Rainbow trout* samples were As, Se, Zn, and Al in stations S2, S3, S4, and S5. The lowest arsenic level in *Rainbow trout* samples was detected at station S1. The highest Cd and Pb levels in *Rainbow trout* were identified in samples collected from stations S6, S7, S8, S9, S10, and S15. Pb and Cd, in particular, are on the hazardous substances priority list of the Comprehensive Environmental Response, Compensation, and Liability Act (CERCLA), administered by the U.S. Environmental Protection Agency (EPA) and the Agency for Toxic Substances and Disease Registry (ATSDR) The highest Cu and Ni levels in *Rainbow trout* were determined at stations S11 and S12. The high concentrations of Cu, Zn, and Pb are reported to be due to the intensive copper mining activities and Zn reserves in this region (Yılmaz Bayrak 2016; Çevik et al. 2008). As seen in Fig. 4, for the water samples, the highest levels of Zn, Ni, and Co were detected at stations S2, S3, S4, and S5. Consequently, the consumption of these metals from existing fish populations may pose a significant threat. Cu, Al, Fe, and Pb were found at the highest levels in the water samples from station S8, whereas the highest levels of As and Hg were detected in samples from station S14. Cd and Mn were found at the highest concentrations in water samples collected from stations S6, S7, S8, and S9 (Fig. 4). Because it is not an essential metal, cadmium is extremely dangerous for fish. It is considered that the source of PTEs detected in *Rainbow trout* and water is the discharge of untreated industrial and domestical wastewater into streams and rivers in the Black Sea region. It is a well-known fact that the use of agricultural chemicals such as pesticides, insecticides, fertilizers, etc., which contain PTEs, due to agricultural production, increases metal pollution in the region. In the study area, which is impacted by global climate change, it is also hypothesized that abrupt floods, water overflows, and landslides brought on by heavy rainfall have exacerbated PTEs pollution, especially affecting stations S6, S7, S8, S9, S10, and S15. Additionally, it is thought that the bioaccumulation of metal contamination in the local ecology has also been facilitated by accidents brought on by continuous mining operations in the Eastern Black Sea Region. Furthermore, it has been suggested that increases in PTEs content brought on by agricultural and fishery operations could be the cause of the observed differences between stations (Figs. 3 and 4).

### Indicators of ecotoxicological and public health risk

BCF (Bioconcentration Factor) values are characterized by the absorption, transfer, and accumulation of contaminants from the dissolved phase. However, the bioconcentration factor (BCF), calculated as the ratio of tissue concentration to water concentration, reflects not only the final tissue concentration but also the dynamics of exposure. When BCF (Bioconcentration Factor) values, which are characterised as the absorption, transfer ability and accumulation of pollutants from the dissolved phase (Peñaloza et al. 2023; Rahman et al. 2023), the highest BCF value was observed at station S11, while the lowest BCF values were found at stations S1, S8 and S14.

A high MPI value indicates a greater cumulative metal accumulation in the fish sample. In this case, the consumption of fish with high MPI values may pose a potential public health risk (Rabiul Islam et al. 2017). Consuming fish with an MPI value > 1 as food can pose a potential health risk (Töre et al. 2021; Kalipci et al. 2023). In this study, based on the concentrations of 14 PTEs detected in all sampled *Rainbow trout* muscle tissues, the calculated MPI level was found to be below 1 (0.78). The MPI results suggest that the consumption of all sampled *Rainbow trout* as food does not pose a potential health risk. In this study, the average MPI level in the muscles of fish species was found to be lower than the values reported in studies conducted in Egypt, Türkiye, Greece, Italy, Spain, Serbia, and Portugal, but higher than those reported in China, Bangladesh, France, Belgium, and some coastal regions of Türkiye (Table S5—See the Supplementary Material File).

**Fig. 5:**
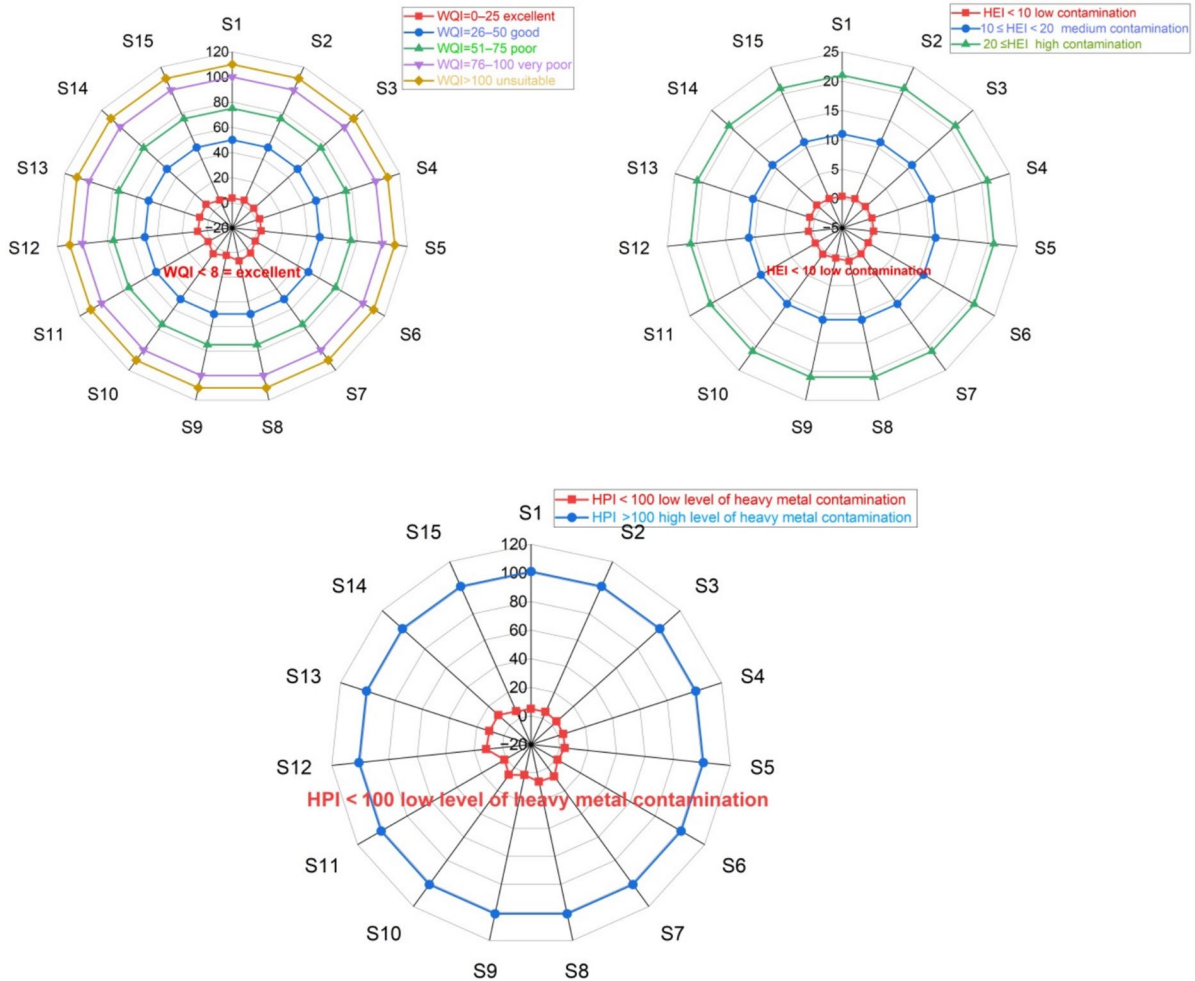
Geospatial distribution of WQI, HEI, HPI in water by radar plots

The summarized geographical distribution of each PTEs detected in the waters where *Rainbow trout* is cultivated, derived from the ecotoxicological index values of WQI, HEI, and HPI, is presented in Fig. 5. In this study, while the WQI index at all stations was classified as excellent water quality, the HEI index was categorized as low pollution and a low level of pollution was detected according to the HPI index, which indicates that there is no significant health risk. These results indicate that the water quality in the 15 fish ponds where *Rainbow trout* is cultivated is at a good level.

**Fig. 6:**
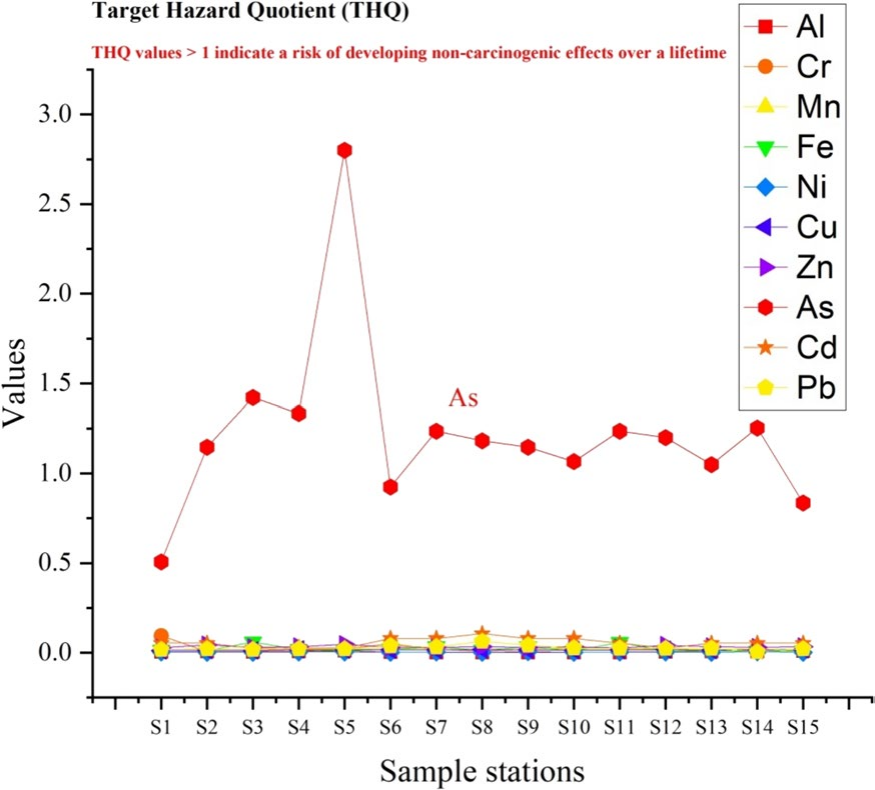
THQ values in Rainbow trout

**Fig. 7:**
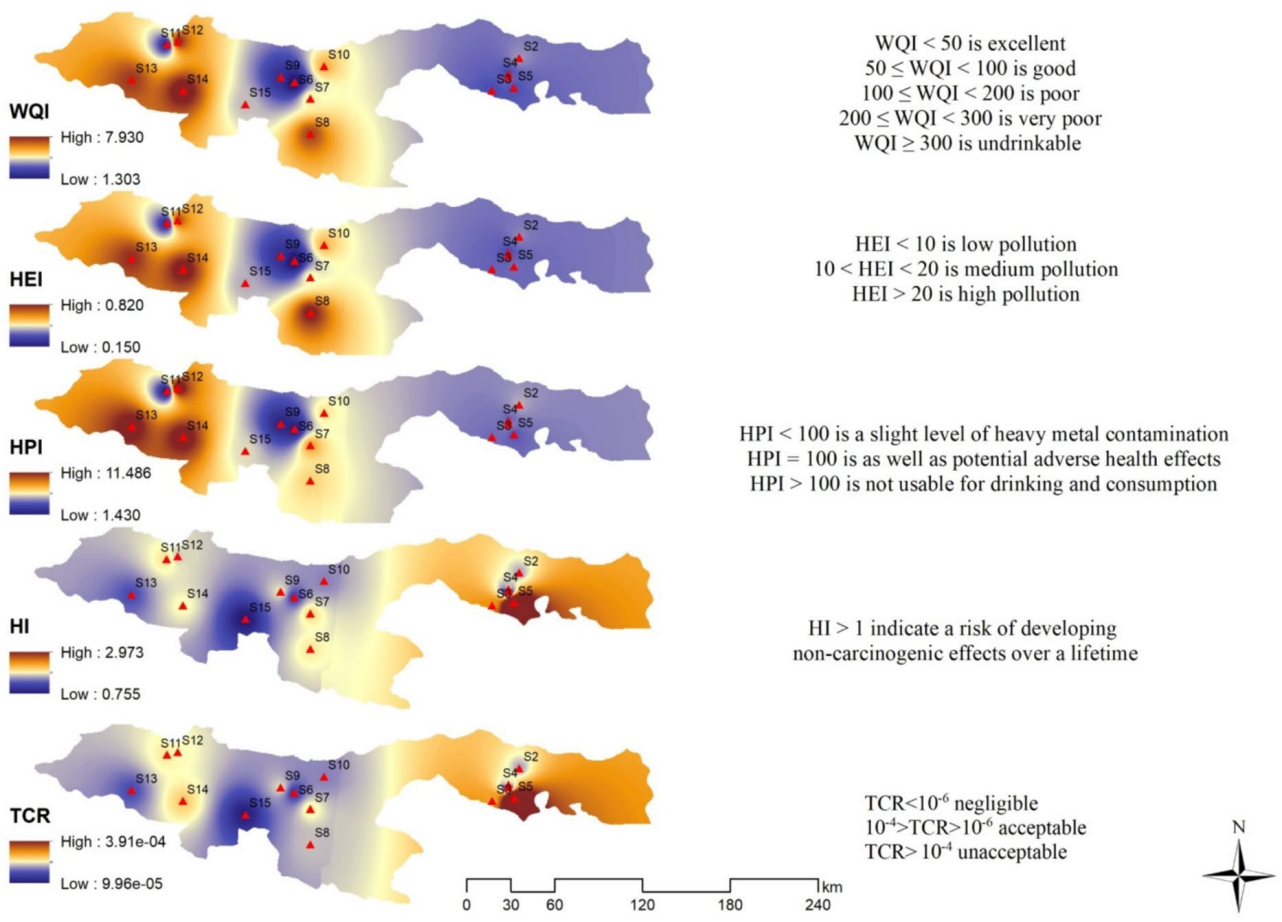
Bioaccumulation distribution of WOI, HEI, HPI, HI, TCR

In view of the fact that the THQ for Al, Fe, Cr, Mn, Ni, Cu, Zn, Cd and Pb in rainbow trout at all stations is less than 1, it is predicted that these PTEs are not associated with the occurrence of non-carcinogenic health consequences. However, the fact that THQ > 1 for As at all stations except S1 and S15 suggests the potential occurrence of non-carcinogenic adverse health effects related to As (Figs. 6 and 7). As a matter of fact, in a study that analysed the severity of health risks of PTEs with different models (Monte Carlo simulations), it was reported that drinking arsenic-contaminated water can cause cancer and children are more susceptible than adults (Rahman et al. 2024). HI values higher than the safe limit indicate that exposure to trace metals in adults and children has a possible health risk (Hasan et al. 2024). The highest HI level was determined at station S5 (2.9). The fact that HI > 1 at all stations except S1 also indicates the potential for non-carcinogenic adverse health effects to occur (Fig. 7).

When the average of the CR values calculated at all stations is considered, the order can be listed as As > Cr > Ni > Cd > Pb. The results indicate that while the cancer risk value for As (0.00017) suggests a significant cancer risk, other potentially carcinogenic metals such as Pb and Cd demonstrate minimal risk. Additionally, the levels of Ni and Cr were found to be within the permissible limits. In terms of the TCR risk level, it was observed that cancer risk due to metal accumulation may exist at all stations except for station S1 (Fig. 7).

**Fig. 8:**
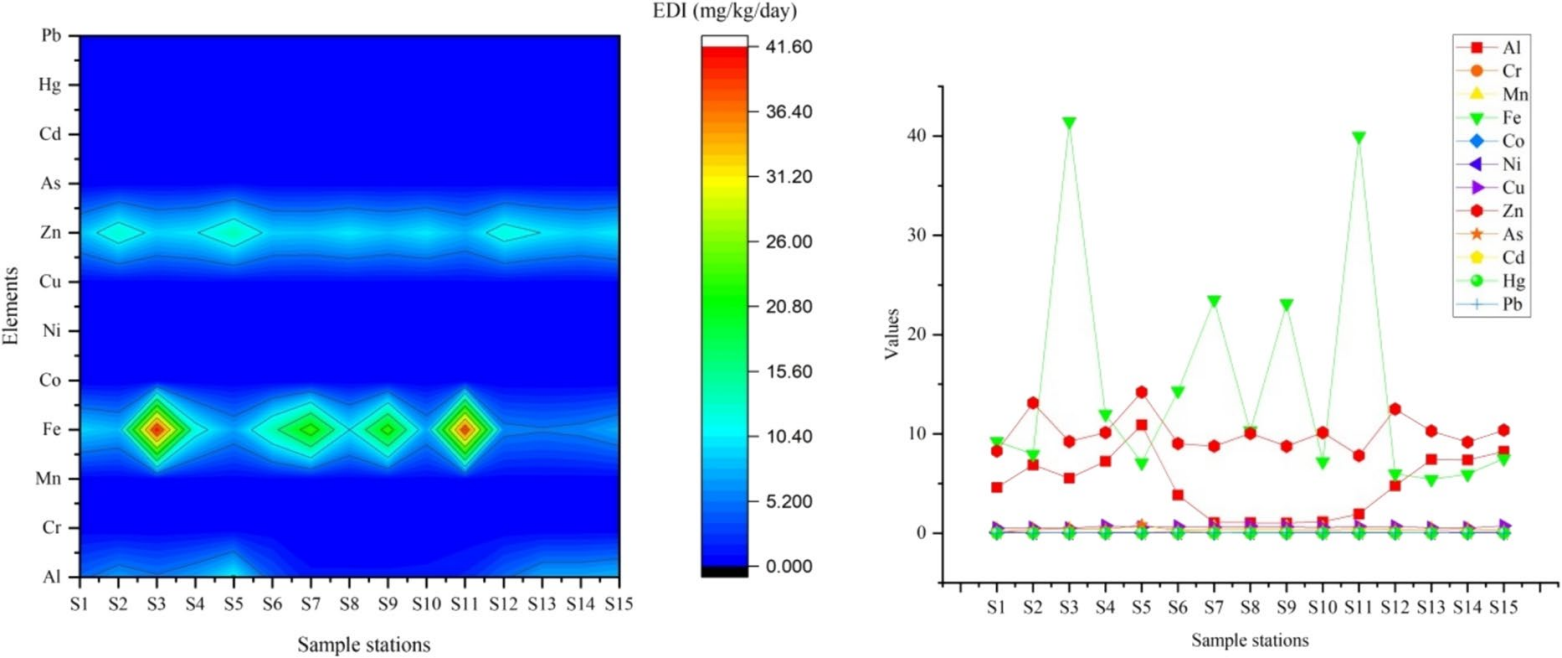
EDI values in Rainbow trout

The EDI values calculated for PTEs resulting from the consumption of *Rainbow trout* by adult humans are presented in Table S6. In this study, the EDI values determined in the analyzed fish samples were found to be significantly above the tolerable daily intake (TDI) limits for As, Cd, Fe, and Zn, indicating that the consumption of the analyzed tissue samples may pose a health risk associated with the intake of PTEs (Fig. 8). This finding corroborates earlier research indicating that toxic metals, including As and Cd, have the potential to induce carcinogenesis. Studies in *Rainbow trout* fry (O. mykiss) have shown that tolerance to chronic As exposure increases with temperature, while the opposite is true in invertebrates (Kumari et al. 2017).

### Analysis outputs of statistical assessment

**Fig. 9:**
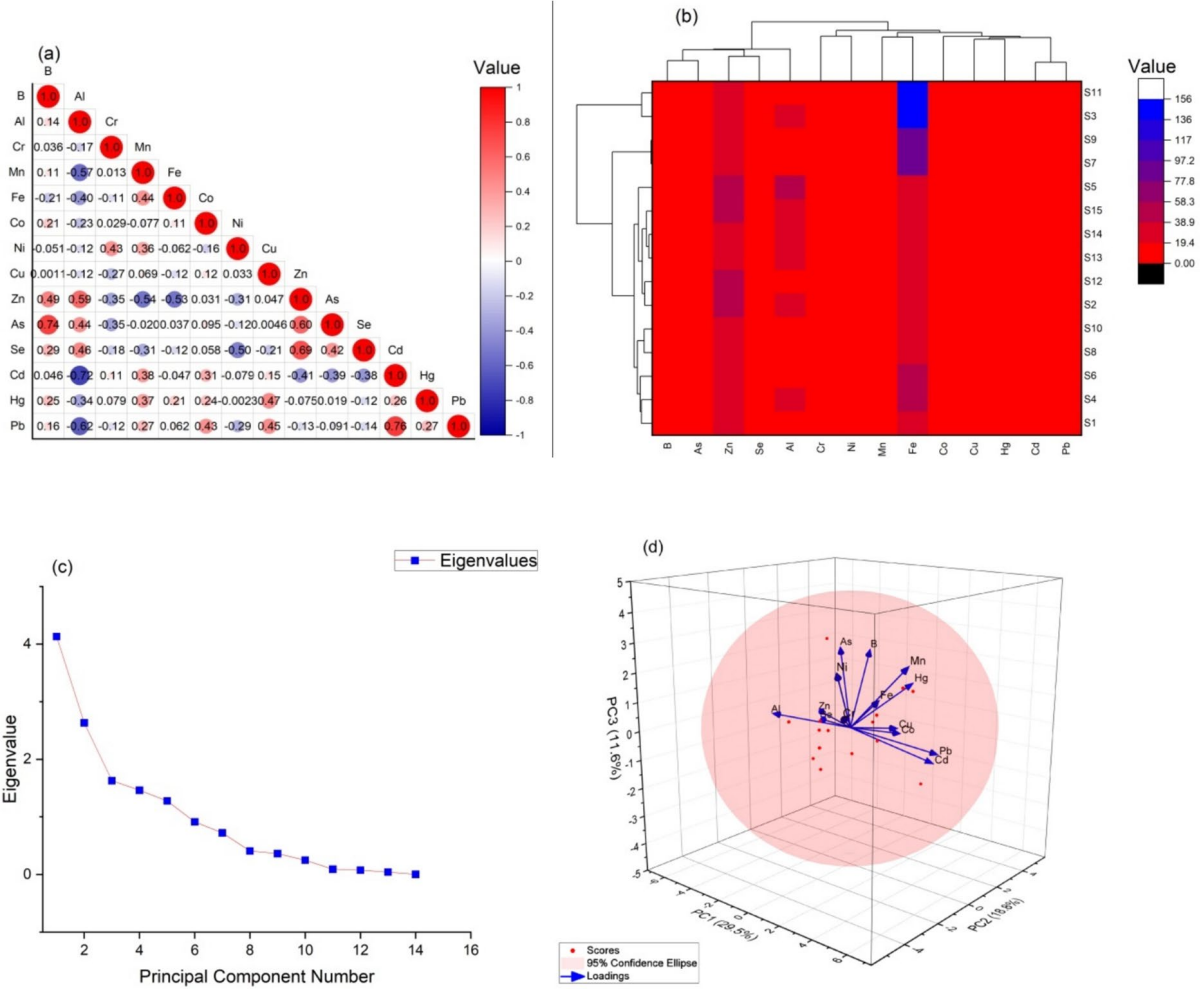
Correlation matrix (**a**), CA heat map (**b**), scree plot of Eigen values (**c**) and PCA (**d**) of PTEs in Rainbow trout

Figure 9a shows the Pearson correlation coefficients of PTEs in *Rainbow trout* sampled from 15 fish farms in the Eastern Black Sea Region of Türkiye. For *Rainbow trout* samples, As and B (r = 0.74), Pb and Cd (0.76), Zn and Al (r = 0.59), Se and Zn (r = 0.69), As and Zn (r = 0.60) were highly correlated. Al, Zn, Se and As were found to have a high correlation with each other. The presence of elements is often attributed to anthropogenic activities; this is evidenced by the positive correlations found between metal concentrations. When two different metals have similar amounts, for example, it indicates that they come from the same source, have the same characteristics, and are dependent on the aquatic environment (Ali et al. 2022).

In this study, the cluster heat map and dendrogram of the fish farms where *Rainbow trout* sampling was carried out were constructed by Ward connectivity approach using Euclidean distance and given in Fig. 9b. Two clusters were visible in the vertical section of the dendrogram: The PCA results indicate that the chosen metals come from similar sources. B, As, Zn, Se, and Al make up Cluster 1, whereas Fe, Mn, Cr, Ni, Co, Cu, Hg, Cd, and Pb make up Cluster 2 (Fig. 9d). Among all stations studied, S1, S4, S6, S8, S10, S2, S12, S13, S14, S15, S5, S7 and S9 were assigned to cluster 1. Cluster 2 included S11 and S3. The two-way cluster analysis (CA) and PCA results were found to be highly compatible with each other and the correlation ratio between PTEs was above 0.70. PCA analyses are used in many investigations to identify potential sources of elements and major effects of metals (Haque et al. 2023; Simsek et al. 2025). Standardized data sets were used for PCA. Together with the interrelationships between variables, an observable characteristic is the existence of an eigenvector with a magnitude larger than one, as shown in Fig. 9d. Three PCs with an eigenvalue larger than 1 were found to be present in the element data according to the PCA results, and they accounted for 59.90% of the variance. PTEs for B, Co, Ni, Zn, As, and Se made up the first PC1, which was responsible for 29.5% of the transition. Although these PTEs are found to be predominantly of terrestrial origin, as shown by the spatial distribution maps (Figs. 2 and 3) and correlation matrix analysis, it is thought that the mining and extraction activities in the region lead to the mixing of PTEs, especially lead, arsenic, mercury, zinc and copper, into soil and water resources. The second principal component, which included Fe, Al, Mn, Cu and Hg was found to account for 18.8% of the total variance (Fig. 9d). PC 3 contains Pb, Cd and Cr PTEs (11.6%).

**Fig. 10:**
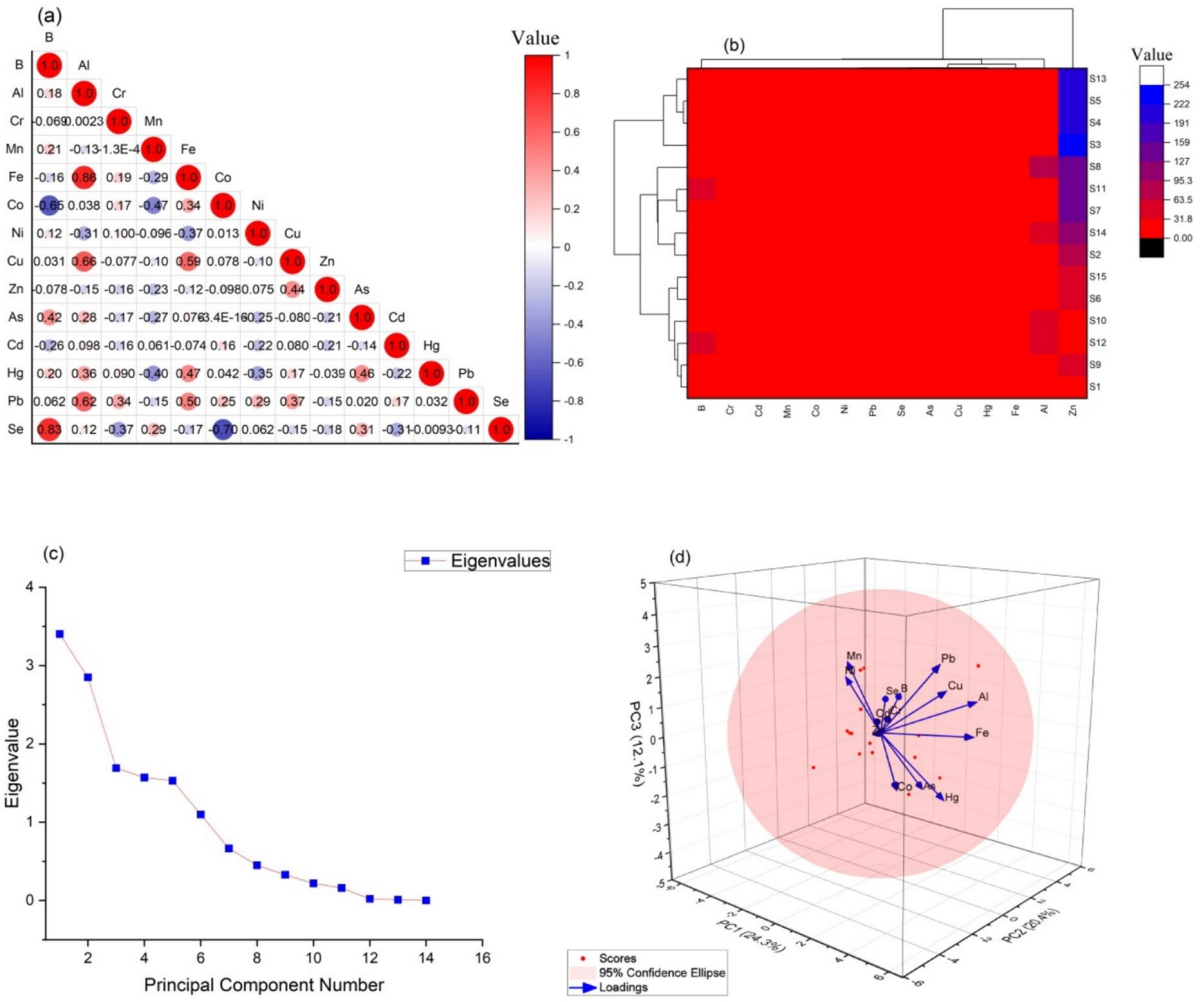
Correlation output (**a**), CA heat graph (**b**), scree plot of Eigen values (**c**) and PCA (**d**) of PTEs in water

Figure 10 shows the Pearson correlation coefficients of PTEs in water sampled from 15 fish farms in the Eastern Black Sea Coastline of Türkiye. For water samples, Fe and Al (r = 0.86), Se and B (0.83), Cu and Al (r = 0.66), Pb and Al (r = 0.62), Fe and Cu (r = 0.59) were highly correlated. Al, Fe, Cu and Pb were found to have a high correlation with each other. Due to the geogenic structure of the region, Fe and Al are the two most abundant elements found. Similar to the fish sampling, the cluster heat map and dendrogram of the fish farms where water sampling was carried out were generated by Ward connectivity approach using Euclidean distance and presented in Fig. 10b. As can be seen in Fig. 10b, the dendrogram presents two clusters in the vertical part: B, Cr, Cd, Mn, Co, Ni, Ni, Pb, Se, As, Cu, Hg, Fe and Al in cluster 1 and only Zn in cluster 2, in agreement with the PCA results of a comparable origin of the selected PTEs (Fig. 10d). Among all stations studied, S3, S4, S5, and S13 were assigned to cluster 1. Cluster 2 included S8, S11, S7, S14, S2, S15, S6, S10, S12, S9, and S1. As shown in Fig. 10d, the PCA matrix confirms the positive and negative correlations between the PTEs. The two-way cluster analysis and PCA results were found to be compatible with each other and there was a high correlation between PTEs. When the PCA results are examined, it is seen that there are 3 factorial groups representing 56.8% of the dataset. It is thought that the PTEs detected in the water samples in the stations are caused by the accumulation of PTEs in the soil of some pesticides and chemical fertilisers used in the agriculture of the region and their leaching into water resources and groundwater levels.

## Conclusion

The present study provides valuable insights into the bioaccumulation patterns and carcinogenic effects of PTEs in *Rainbow trout* and pond waters from fish farms located in the Eastern Black Sea Region, highlighting the ecotoxicological and health risks associated with these pollutants. Furthermore, this research offers a multivariate statistical analysis-based dataset and, for the first time, presents a PTEs distribution map and ecological risk index map for the region, serving as a reference for future studies by other researchers working in the area and as a resource for policymakers.

When the findings of this study are holistically evaluated, it is evident that the consumption of *Rainbow trout* may pose potential health risks. The THQ and HI index results for fish muscle tissue indicate the potential for non-carcinogenic adverse health effects related to As at all stations except S1. Additionally, the EDI, CR, and TCR levels suggest the potential for cancer risk arising from metal accumulation at all stations. The indices and ecological indicators used in this study point to a cancer risk specifically associated with As through the consumption of Rainbow trout. Because fish are constantly exposed to water pollution, they can convert the toxic form of As into a less toxic form. The ability of fish to absorb As from their food and water is clearly present. The bioavailability and absorption processes of arsenic in the aquatic environment have an impact on the buildup of As in muscle tissue. Indeed, it was also determined that the As and Mn levels in fish muscle tissue exceeded the maximum permissible limits (MPLs). The results of correlation, PCA, and cluster analyses suggest that PTEs contamination in fish may arise from similar environmental sources or similar geochemical behaviour of the PTEs, and that anthropogenic activities are a common source of these elements. The HEI and HPI ecotoxicological index values for the waters in which *Rainbow trout* are raised indicate low levels of PTEs contamination. While high levels of PTEs pollution were not detected in the ponds in the study area, a lack of awareness among local agricultural communities, the discharge of untreated wastewater into receiving bodies, and uncontrolled waste management are expected to lead to localized increases in PTEs pollution. Furthermore, the increasing number of mining accidents in the region due to increased mining activity and the risk of floods and landslides raises the risk of PTEs pollution to alarming levels and constitutes the most serious threat requiring action.

## Supplementary Information

Below is the link to the electronic supplementary material.

Supplementary file1 (DOCX 63 kb)

## Data Availability

All data supporting the findings of this study are available within the paper and its Supplementary Information.
