# Supplementary material for: Mapping geochemical distribution, toxicity and ecological risk indices of potentially toxic elements in cultured fish and surface water (Blacksea catchment/Türkiye)
Source: Biometals. 2026 Jan 20;39(2):775–94. doi: 10.1007/s10534-025-00785-4 (PMC13083499; doi:10.1007/s10534-025-00785-4)
Supplement: Supplementary file 1 — Supplementary file1 (DOCX 63 kb) [file 10534_2025_785_MOESM1_ESM.docx]

**Supplementary Material File**

**Mapping geochemical distribution, toxicity and ecological risk indices of potentially toxic elements in cultured fish and surface water (Blacksea catchment/ Türkiye)**

Mustafa TÜRKMEN^a^, Erkan KALIPCI^b^, Mehmet Ali DERELİ^b*^, Hüseyin CÜCE^c^, Aysun TÜRKMEN^d^

**1.Analysis of Health Risk Indexes**

**1.1. Assessment of Metal Pollution Index (MPI)**

The Metal Pollution Index is a mathematical model that epitomize the value for all metals in a single form. MPI is a credible and accurate index to monitor PTEs contamination in the food and the aquatic ecosystem. In this study; MPI was calculated following the equation proposed by Usero et al. (1997):

MPI = (Cf_1_xCf_2_xCf_3_x … xCf_n_)^1/n^ (1)

where: Cf_i_: is the mean concentration of metal i in the samples, n: total number of metals.

**1.2.** **Bioconcentration Factor (BCF)**

The bioconcentration factor (BCF) is characterized as the absorption of contaminants from the dissolved phase (Peñaloza et al., 2023) and can be calculated using the following equation:

$$BCF=\frac{C}{C_{W}} (2)$$

where C represents the concentrations of contaminants in organisms (mg/kg) at equilibrium, and Cw is the concentration of contaminants in the water (mg/L).

**1.3. Risk Assessment for Consumption of Rainbow Trout**

The non-carcinogenic risk, Estimated Weekly Intake (EWI), Total Hazard Quotient (THQ), and Hazard Index (HI) values were calculated according to the equations described by Simukoko (Schiel and Rienitz, 2011; Peñaloza et al., 2023). The Target Hazard Quotient is the ratio between the Estimated Daily Intake (EDI, mg/kg/day) of the metals, defined as the absorption dose multiplied by the absorption efficiency (Arm) of the metals in the human gastrointestinal tract (Yu et al., 2014), and the Oral Reference Dose (RfD, mg/kg body weight/day). The values of EWI and EDI (Estimated Daily Intake) for each element were compared with the Provisional Tolerable Weekly Intake (PTWI) and PTDI (Permissible Tolerable Daily Intake) established by the World Health Organization (WHO) (Bat et al., 2015). The Target Hazard Quotient is the ratio between the Estimated Daily Intake (EDI) and the Oral Reference Dose (RfD, mg/kg body weight/day) (Mwakalapa et al., 2019; Peñaloza et al., 2023), where the RfD is the level or dose of daily exposure (usually expressed in milligrams of toxic chemical substance per kilogram of body weight per day) for the human population (Simukoko et al., 2021). Finally, the Hazard Index (HI), also referred to as the Total Hazard Quotient (THQ), is derived from the summation of individual THQ values corresponding to the metals. THQ and HI values > 1 indicate a risk of developing non-carcinogenic effects over a lifetime (Opresko et al., 1998).

$$EDI=\frac{MC\times IR\times EF\times ED}{BW\times AT} \times ADAF \times AR_{m} (3)$$

where MC = mean metal concentration, IR = acceptable ingestion rate (0.34 kg/person/day), EF = exposure frequency (365 days/year), ED = exposure duration (74.8 years, which is the expected average life time), BW = average body weight (60 kg for an adult), AT = average exposure time for non-carcinogenic element (EF × ED), and ADAF = age-dependent adjustment factor (adult:1) (Saha et al., 2021). The value of ARm for Pb is 33% and for As is 75% (USEPA, 2005).

$$THQ=\frac{E_{F}\times ED\times FIR\times C}{RfD\times W_{AB}\times TA}\times{10}^{-3} (4)$$

where E_F_ = exposure frequency, ED = exposure duration, FIR = fish ingestion rate, C = mean metal concentration, RfD = oral reference dose, W_AB_ = average body weight of an adult, and TA = average exposure time with non-carcinogenic effect (E_F_ ∗ ED).

$$HI=\sum_{i=1}^{n} {THQ}_{i} (5)$$

The carcinogenic risk (CR) was evaluated to assess the possibility of cancer occurrence in individuals over their lifetime due to exposure to carcinogenic agents. The acceptable range of carcinogenic risk is from 10^−4^ to 10^−6^ , and CR values higher than 10^−4^ are likely to increase the probability of carcinogenic hazard impact (Wang et al., 2020). The CR of arsenic and lead was calculated using Equation (6):

$$CR=\frac{E_{F}\times ED\times CSF\times EDI}{TA} \times{10}^{-3} (6)$$

where CSF = cancer slope factor of cancer-causing agents (mg/kg-day)^−1^ which, for this study, was available for Pb (0.0085 mg/kg-day)^−1^ and As (1.5 mg/kg-day)^−1^ (Peñaloza et al., 2023) according to the database of the Integrated Risk Information System USEPA; EDI = estimated daily intake (EWI/7), while the total cancer risk (TCR) due to the consumption of rainbow trout from the evaluated fish farms was calculated as the sum of individual cancer risks (Schiel and Rienitz, 2011; Peñaloza et al., 2023), using Equation (7).

$$TCR=\sum_{i=1}^{n} {CR}_{i} (7)$$

2. **PTEs Values in Water and Rainbow Trout Samples:** The average concentrations of PTEs detected in *Rainbow trout* widely consumed and economically valuable and in water samples collected from 15 pond farms located in the Eastern Black Sea Region of Türkiye are presented in Table S1 and Table S2 below.

**Table S1.** Recovery rates of PTEs detected in the certified reference material used (TORT-2 and UME CRM 1201).

| PTEs | Recovery (%) (TORT-2) | Recovery (%) (UME CRM 1201) |
| --- | --- | --- |
| As | 98.2 | 94.0 |
| Cd | 99.4 | 99.6 |
| Co | 94.6 | 99.7 |
| Cr | 95.2 | 98.1 |
| Cu | 97.5 | 98.9 |
| Fe | 97.0 | 99.9 |
| Mn | 99.1 | 99.1 |
| Ni | 99.4 | 99.5 |
| Pb | 98.9 | 99.5 |
| Al | 94.0 | 95.6 |
| Zn | 96.5 | 99.5 |
| Hg | 98.7 | 98.9 |
| Se | 99.1 | 99.0 |

**Table S2.** PTEs mean concentrations in fish samples (mg/kg wet weight).

| PTEs | Sample Stations | | | | | | | | | | | | | | |  |
| --- | --- | --- | --- | --- | --- | --- | --- | --- | --- | --- | --- | --- | --- | --- | --- | --- |
|  | **S1** | **S2** | **S3** | **S4** | **S5** | **S6** | **S7** | **S8** | **S9** | **S10** | **S11** | **S12** | **S13** | **S14** | **S15** | **Mean** |
| B | 0.23 | 0.29 | 0.21 | 0.15 | 2.42 | 0.57 | 0.54 | 0.59 | 0.92 | 1.3 | 0.11 | 0.27 | 0.17 | 0.23 | 0.23 | **0.55** |
| Al | 17.3 | 25.8 | 20.8 | 27.12 | 40.9 | 14.4 | 3.98 | 3.95 | 3.89 | 4.31 | 7.25 | 17.9 | 27.9 | 27.7 | 30.9 | **18.2** |
| Cr | 1.06 | 0.07 | 0.08 | 0.11 | 0.25 | 0.56 | 0.23 | 0.10 | 0.22 | 0.33 | 0.25 | 0.22 | 0.06 | 0.27 | 0.08 | **0.26** |
| Mn | 1.74 | 1.65 | 1.75 | 1.90 | 1.74 | 1.84 | 2.20 | 1.71 | 2.24 | 1.89 | 1.96 | 1.63 | 1.82 | 1.80 | 1.64 | **1.83** |
| Fe | 34.7 | 29.8 | 155.5 | 44.9 | 26.6 | 53.8 | 88.1 | 38.7 | 86.7 | 27.0 | 150 | 22.4 | 20.3 | 22.2 | 28.2 | **55.2** |
| Co | 0.03 | 0.02 | 0.02 | 0.01 | 0.03 | 0.01 | 0.03 | 0.04 | 0.02 | 0.02 | 0.03 | 0.02 | 0.02 | 0.01 | 0.03 | **0.02** |
| Ni | 0.37 | 0.15 | 0.14 | 0.28 | 0.23 | 0.18 | 0.28 | 0.16 | 0.38 | 0.22 | 0.28 | 0.32 | 0.14 | 0.42 | 0.22 | **0.25** |
| Cu | 1.9 | 1.98 | 1.84 | 2.79 | 2.28 | 2.51 | 2.33 | 2.64 | 2.42 | 2.20 | 2.45 | 2.60 | 2.04 | 2.01 | 2.80 | **2.32** |
| Zn | 31.0 | 49.2 | 34.6 | 38.0 | 53.3 | 33.9 | 32.9 | 37.7 | 32.8 | 38.0 | 29.3 | 46.9 | 38.6 | 34.4 | 38.9 | **37.9** |
| As | 0.57 | 1.29 | 1.60 | 1.50 | 3.15 | 1.04 | 1.39 | 1.33 | 1.29 | 1.20 | 1.39 | 1.35 | 1.18 | 1.41 | 0.94 | **1.38** |
| Se | 0.75 | 1.32 | 0.81 | 0.90 | 1.12 | 0.66 | 0.65 | 0.65 | 0.62 | 0.70 | 0.67 | 0.66 | 0.80 | 0.40 | 0.66 | **0.76** |
| Cd | 0.02 | 0.02 | 0.01 | 0.01 | 0.01 | 0.03 | 0.03 | 0.04 | 0.03 | 0.03 | 0.02 | 0.01 | 0.02 | 0.02 | 0.02 | **0.02** |
| Hg | 0.07 | 0.07 | 0.07 | 0.07 | 0.08 | 0.09 | 0.10 | 0.07 | 0.08 | 0.08 | 0.08 | 0.08 | 0.06 | 0.06 | 0.09 | **0.08** |
| Pb | 0.09 | 0.11 | 0.09 | 0.11 | 0.11 | 0.20 | 0.17 | 0.33 | 0.22 | 0.14 | 0.13 | 0.12 | 0.13 | 0.03 | 0.11 | **0.14** |

**Table S3.** PTEs mean concentrations in water samples (µg/l).

| PTEs | Sample Stations | | | | | | | | | | | | | | |  |
| --- | --- | --- | --- | --- | --- | --- | --- | --- | --- | --- | --- | --- | --- | --- | --- | --- |
|  | **S1** | **S2** | **S3** | **S4** | **S5** | **S6** | **S7** | **S8** | **S9** | **S10** | **S11** | **S12** | **S13** | **S14** | **S15** | **Mean** |
| B | 17.9 | 19.2 | 17.0 | 22.6 | 19.7 | 9.0 | 19.7 | 18.9 | 22.5 | 19.5 | 36.9 | 35.7 | 17.1 | 26.0 | 18.0 | **21.3** |
| Al | 22.9 | 23.4 | 18.8 | 19.2 | 22.9 | 9.79 | 17.8 | 74.3 | 29.0 | 50.0 | 26.8 | 33.0 | 31.7 | 44.1 | 24.9 | **29.9** |
| Cr | 0.12 | 0.10 | 0.09 | 0.11 | 0.11 | 0.02 | 0.08 | 0.12 | 0.13 | 0.03 | 0.01 | 0.13 | 0.06 | 0.06 | 0.26 | **0.10** |
| Mn | 0.21 | 0.08 | 0.16 | 0.28 | 0.08 | 0.27 | 0.36 | 0.23 | 0.50 | 0.12 | 0.31 | 0.20 | 0.03 | 0.19 | 0.17 | **0.21** |
| Fe | 4.62 | 3.6 | 3.82 | 0.29 | 4.99 | 2.67 | 1.76 | 22.0 | 7.35 | 11.4 | 0.25 | 6.70 | 14.9 | 10.8 | 9.93 | **7.01** |
| Co | 0.21 | 0.22 | 0.20 | 0.21 | 0.22 | 0.22 | 0.19 | 0.21 | 0.21 | 0.21 | 0.18 | 0.20 | 0.22 | 0.21 | 0.21 | **0.21** |
| Ni | 0.22 | 0.52 | 0.20 | 0.22 | 0.16 | 0.17 | 0.11 | 0.16 | 0.18 | 0.02 | 0.33 | 0.16 | 0.16 | 0.10 | 0.21 | **0.19** |
| Cu | 0.63 | 0.55 | 0.92 | 0.86 | 0.76 | 0.36 | 0.46 | 1.62 | 0.67 | 0.55 | 0.60 | 0.68 | 0.95 | 0.69 | 0.21 | **0.70** |
| Zn | 23.8 | 90.2 | 253.6 | 212.9 | 209.5 | 52.1 | 152.1 | 134.8 | 32.3 | 24.8 | 145.7 | 22.5 | 199.2 | 98.6 | 54.4 | **113.7** |
| As | 0.26 | 0.16 | 0.08 | 0.21 | 0.24 | 0.09 | 0.08 | 0.10 | 0.02 | 0.32 | 0.20 | 0.35 | 0.10 | 0.66 | 0.13 | **0.20** |
| Se | 0.08 | 0.10 | 0.12 | 0.07 | 0.09 | 0.11 | 0.13 | 0.10 | 0.20 | 0.19 | 0.48 | 0.29 | 0.11 | 0.24 | 0.11 | **0.16** |
| Cd | 0.05 | 0.05 | 0.05 | 0.05 | 0.05 | 0.05 | 0.05 | 0.05 | 0.05 | 0.06 | 0.04 | 0.05 | 0.04 | 0.04 | 0.04 | **0.05** |
| Hg | 0.97 | 0.94 | 0.80 | 0.63 | 0.60 | 0.12 | 1.79 | 1.36 | 0.16 | 1.23 | 0.04 | 2.74 | 2.65 | 2.44 | 1.16 | **1.79** |
| Pb | 0.23 | 0.46 | 0.24 | 0.24 | 0.35 | 0.19 | 0.25 | 0.48 | 0.34 | 0.32 | 0.25 | 0.30 | 0.22 | 0.31 | 0.33 | **0.30** |

**Table S4.** Calculations of applied ecotoxicological risk assessment indices in water.

| **Index** | **Formula** | **Formula Explanations** | **Water Quality Assessment** | **Standard Used** | **Reference** |
| --- | --- | --- | --- | --- | --- |
| **WQI** | 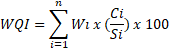  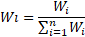 | W_I_: Relative weight  W_i_:Values assigned to i (1-5)  C_i_: Values of measured parameters  S_i_: Standard value | <50: Excellent water quality  50–100: Good water quality  100–200: Poor water quality  200–300: Very poor water quality  >300: Not available | (TSWQR 2021) | (Xiao et al., 2019) |
| **HPI** | 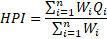  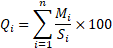 | Q_i_: Subindex of toxic substance  W_i_: Unit load of parameter i  M_i_: Values of toxic substance  S_i_: Standard value | <45: Low pollution  45-90: Medium pollution  >90: High pollution | (WHO 2011) | (Mohan et al., 1996) |
| **HEI** | 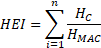 | H_c_: Observed value of parameters  H_mac_: Limit value of parameters | <10: Low pollution  10-20: Medium pollution  >20: High pollution | (WHO 2011) | (Edet and Offiong, 2002) |

**Table S5.** Comparison of MPI levels in fish identified in different countries (Adapted from Kalıpcı et al., 2023).

| Country | MPI Value | References |
| --- | --- | --- |
| Türkiye | 0.78 | **This study** |
| Egypt | 2.61 | Abdel-Khalek et al. (2020) |
| Türkiye | 1.33 | Töre et al. (2021) |
| Greece | 1.87 | Castritsi-Catharios et al. (2015) |
| İtaly | 5.84 | Carpene et al. (1998) |
| Spain | 2.11 | Vicente-Martorell et al. (2009) |
| Serbia | 1.92 | Milošković et al. (2016) |
| Portugal | 2.38 | Lourenço et al. (2012) |
| China | 0.29 | Jia et al. (2017) |
| Bangladesh | 0.77 | Vicente-Martorell et al. (2009) |
| France | 0.67 | Schnitzler et al. (2011) |
| Belgium | 0.77 | Schnitzler et al. (2011) |
| Türkiye | 0.27 | Kalipci et al. (2023) |
| Türkiye | 0.09 | Ustaoğlu and Yüksel (2024) |

**Table S6.** Comparison of EDI values from *Rainbow trout* consumption with standard tolerable daily intake (TDI).

| PTEs | EDI (mg/kg/day) | TDI (mg/kg/day) |
| --- | --- | --- |
| As | 0.366756 | 0.00214 (JECFA, 1989) |
| Cd | 0.005689 | 0.0008 (JECFA, 2011) |
| Co | 0.006044 | 0.003 (Finley et al., 2012) |
| Cr | 0.069156 | 0.3 (EFSA, 2014) |
| Cu | 0.618489 | 0.5 (JECFA, 1982) |
| Fe | 14.736 | 0.8 (JECFA, 1983) |
| Mn | 0.489067 | 0.14 (USEPA, 2016) |
| Ni | 0.067022 | 0.0012 (WHO, 2011) |
| Pb | 0.037156 | 0.0015 (EFSA, 2010) |
| Zn | 10.12444 | 0.3 (JECFA, 1982) |

**References**

Usero J, Gonza´lez-Regalado E, Gracia I (1997). Trace metals in the bivalve molluscs Ruditapes decussatus and Ruditapes philippinarum from the atlantic coast of Southern Spain. *Environ Int,* 23:291–298.

Peñaloza, R., Custodio, M., Cacciuttolo, C., Chanamé, F., Cano, D., & Solorzano, F. (2023). Human health risk assessment for exposure to heavy metals via dietary intake of rainbow trout in the influence area of a smelting facility located in Peru. *Toxics*, *11*(9), 764.

Schiel, D.; Rienitz, O. Final report on CCQM-K70: Determination of Hg in natural water at a concentration level required by the European environmental quality standard (EQS). Metrologia (2011), 48, 08011.

Yu, Y.; Wang, X.; Yang, D.; Lei, B.; Zhang, X.; Zhang, X. Evaluation of human health risks posed by carcinogenic and noncarcinogenic multiple contaminants associated with consumption of fish from Taihu Lake, China. Food Chem. Toxicol. 2014, 69, 86–93.

Bat, L.; Sezgin, M. Heavy Metal Levels in Some Commercial Fish from Sinop Coast of the Black Sea, Turkey. In Proceedings of the Twelfth International Conference on the Mediterranean Coastal Environment MEDCOAST, Varna, Bulgaria, 6–10 October 2015.

Mwakalapa, E.B.; Simukoko, C.K.; Mmochi, A.J.; Mdegela, R.H.; Berg, V.; Müller, M.H.B.; Lyche, J.L.; Polder, A. Heavy metals in farmed and wild milkfish (Chanos chanos) and wild mullet (Mugil cephalus) along the coasts of Tanzania and associated health risk for humans and fish. Chemosphere 2019, 224, 176–186.

Simukoko, C.K.; Mwakalapa, E.B.; Bwalya, P.; Muzandu, K.; Berg, V.; Mutoloki, S.; Polder, A.; Lyche, J.L. Assessment of heavy metals in wild and farmed tilapia (Oreochromis niloticus) on Lake Kariba, Zambia: Implications for human and fish health. Food Addit. Contam. 2021, 39, 74–91.

Opresko, D.M.; Young, R.A.; Faust, R.A.; Talmage, S.S.; Watson, A.P.; Ross, R.H.; Davidson, K.A.; King, J. Chemical warfare agents: Estimating oral reference doses. Rev. Environ. Contam. Toxicol. 1998, 156, 1–183.

Saha, S.; Reza, A.H.M.S.; Roy, M.K. Arsenic geochemistry of the sediments of the shallow aquifer and its correlation with the groundwater, Rangpur, Bangladesh. Appl. Water Sci. 2021, 11, 166.

US Environmental Protection Agency (USEPA). Supplemental Guidance for Assessing Susceptibility from Early-Life Exposure to Carcinogens; US Environmental Protection Agency (USEPA): Washington, DC, USA, 2005

Wang, J.; Shan, Q.; Liang, X.; Guan, F.; Zhang, Z.; Huang, H.; Fang, H. Levels and human health risk assessments of heavy metals in fish tissue obtained from the agricultural heritage rice-fish-farming system in China. J. Hazard. Mater. 2020, 386, 121627.

Edet AE, Offiong OE (2002) Evaluation of water quality pollution indices for heavy metal contamination monitoring. A study case from Akpabuyo-Odukpani area, Lower Cross River Basin (southeastern Nigeria) GeoJournal 57:295-304.

EPA (Environmental Protection Agency), 2004. Risk Assessment Guidance for Superfund Volume I Human Health Evaluation Manual (Part A). DOI: EPA/540/1-89/002.

Mohan SV, Nithila P, Reddy SJ (1996) Estimation of heavy metals in drinking water and development of heavy metal pollution index Journal of Environmental Science & Health Part A 31:283-289.

TSWQR (2021) (Turkish Surface Water Quality Regulation) (2021). Turkish Surface Water Quality Regulation. Turkey.

WHO (2011) (World Health Organization) Evaluation of Certain Food Additives and Contaminants: Seventy-Third Report of the Joint FAO/WHO Expert Committee on Food Additives.

Xiao J, Wang L, Deng L, Jin Z (2019) Characteristics, sources, water quality and health risk assessment of trace elements in river water and well water in the Chinese Loess Plateau Science of The Total Environment 650:2004-2012.

Abdel-Khalek, A. A., Zayed, H. S., Elsayad, S. M., & Zaghloul, K. H. (2020). Assessment of metal pollution impacts on Tilapia zillii and Mugil cephalus inhabiting Qaroun and Wadi El-Rayan lakes, Egypt, using integrated biomarkers. *Environmental Science and Pollution Research*, *27*(21), 26773-26785.

Kalıpcı, E., Cüce, H., Ustaoğlu, F., Dereli, M. A., Türkmen, M. Toxicological health risk analysis of hazardous trace elements accumulation in the edible fish species of the Black Sea in Türkiye using multivariate statistical and spatial assessment. *Environmental Toxicology and Pharmacology*, 97, 104028 (2023).

Töre, Y., Ustaoğlu, F., Tepe, Y., Kalipci, E. (2021). Levels of toxic metals in edible fish species of the Tigris River (Turkey); Threat to public health. *Ecological Indicators*, *123*, 107361.

Castritsi-Catharios, J., Neofitou, N., Vorloou, A.A. (2015). Comparison of heavy metal concentrations in fish samples from three fish farms (Eastern Mediterranean) utilizing antifouling paints. *Toxicol. Environ. Chem.,* **97**, 116–123.

Carpene, E., Martin, B., Dalla, L.L. (1998). Biochemical differences in lateral muscle of wild and farmed gilthead sea bream (series Sparus aurata L.). *Fish. Physiol. Biochem.*, **19**, 229-238.

Vicente-Martorell, J.J., Galindo-Riano, M.D., García-Vargas, M., Granado-Castro, M.D., 2009. Bioavailability of heavy metals monitoring water, sediments and fish species from a polluted estuary. J. Hazard. Mater. 162, 823–836.

Milošković, A., Dojčinović, B., Kovačević, S., Radojković, N., Radenković, M., Milošević, D., & Simić, V. (2016). Spatial monitoring of heavy metals in the inland waters of Serbia: a multispecies approach based on commercial fish. *Environmental Science and Pollution Research*, *23*, 9918-9933.

Lourenço, H.M., Afonso, C., Anacleto, P., Martins, M.F., Nunes, M.L., Lino, A.R. (2012). Elemental composition of four farmed fish produced in Portugal. *Int. J. Food Sci. Nutr.*, 63, 853–859.

Jia, Y., Wang, L., Qu, Z., Wang, C., Yang, Z. Effects on heavy metal accumulation in freshwater fishes: species, tissues, and sizes. *Environmental Science and Pollution Research*, 24, 9379-9386 (2017).

Schnitzler, J.G., Thom´e, J.P., Lepage, M., Das, K. (2011). Organochlorine pesticides, polychlorinated biphenyls and trace elements in wild European sea bass (Dicentrarchus labrax) of European estuaries. *Sci. Total Environ.,* 409, 3680–3686 .

Ustaoğlu, F., Yüksel, B. (2024). Bioaccumulation of metals in muscle tissues of economically important fish species from black sea Lagoon lakes in Türkiye: Consumer health risk and nutritional value assessment. *Microchemical Journal*, 111337.

JECFA (Joint FAO/WHO Expert Committee on food Additives), (1982). Evaluation of certain food additives and contaminants. Twenty-sixth Report of the Joint FAO/WHO Expert Committee on Food Additives. WHO technical Report Series, no 683. World Health Organization, Geneva.

JECFA (Joint FAO/WHO Expert Committee on Food Additives), (1983). Evaluation of certain food additives and contaminants. Twenty-seventh Report of the Joint FAO/WHO Expert Committee on Food Additives. WHO Technical Report Series, No 696. World Health Organization, Geneva.

JECFA (Joint FAO/WHO Expert Committee on Food Additives), (1989). Evaluation of certain food additives and contaminants. Thirty-third Report of the Joint FAO/WHO Expert Committee on Food Additives. WHO Technical Report Series, No 776. World Health Organization, Geneva.

JECFA (Joint FAO/WHO Expert Committee on Food Additives), (2011). Evaluation of certain food additives and contaminants. Seventy-third Report of the Joint FAO/WHO Expert Committee on Food Additives. WHO Technical Report Series, No 960. World Health Organization, Geneva.

Finley, B.L., Monnot, A.D., Paustenbach, D.J., Gaffney, S.H., (2012). Derivation of a chronic oral reference dose for cobalt. Regul. Toxicol. Pharmacol. 64, 491–503.

EFSA (European Food Safety Authority), (2010). Scientific Opinion on lead in food. EFSA J. 8 (4), 1570.

EFSA (European Food Safety Authority), (2014). Scientific opinion on dietary reference values for chromium. EFSA J. 12 (10), 3845.

USEPA (U.S. Environmental Protection Agency), (2016). Integrated Risk Information System. https://www.epa.gov/iris/ (accessed 14.10.16).

WHO (World Health Organization), (2011). Guidelines for Drinking Water Quality. fourth ed. World Health Organization, Geneva.
